# Association of osteoarthritis risk factors with knee and hip pain in a population-based sample of 29–59 year olds in Denmark: a cross-sectional analysis

**DOI:** 10.1186/s12891-018-2183-7

**Published:** 2018-08-21

**Authors:** Joyce A. C. van Tunen, George Peat, Alessio Bricca, Lars B. Larsen, Jens Søndergaard, Trine Thilsing, Ewa M. Roos, Jonas B. Thorlund

**Affiliations:** 1 Department of Sports Science and Clinical Biomechanics, University of Southern Denmark, Odense, Denmark; 2 Research Institute for Primary Care & Health Sciences, Arthritis Research UK Primary Care Centre, Keele University, Staffordshire, UK; 3 Research Unit for General Practice, University of Southern Denmark, Odense, Denmark

**Keywords:** Knee, Hip, Pain, Osteoarthritis, Prevalence, Risk factors

## Abstract

**Background:**

This study aimed to a) describe the prevalence of knee and hip osteoarthritis risk factors in a population of 29–59 year old individuals, b) estimate the association between persistent knee/hip pain and osteoarthritis risk factors, and c) describe the prevalence of osteoarthritis risk factors, including specific biomechanical risk factors, in individuals with prolonged persistent knee or hip pain.

**Methods:**

Participants completed the “Early Detection and Prevention” pilot study questionnaire, including items on presence of knee/hip pain within the last month and osteoarthritis risk factors. Individuals reporting knee/hip problems completed a second questionnaire, including items about most problematic joint and specific biomechanical osteoarthritis risk factors. After describing the prevalence of persistent knee/hip pain and osteoarthritis risk factors among respondents stratified for sex and age, logistic regression was used to estimate the strength of associations between osteoarthritis risk factors and presence of knee/hip pain. The prevalence of prolonged persistent pain (i.e. knee/hip pain reported at both questionnaires) and osteoarthritis risk factors among respondents with prolonged persistent knee and hip pain, were described.

**Results:**

Two thousand six hundred sixty-one respondents completed the first survey. The one-month prevalence of persistent knee/hip pain was 27%. Previous knee/hip injury was associated with persistent knee/hip pain for both sexes in all age groups, while a family history of osteoarthritis was associated with persistent knee/hip pain in all age groups except for 29–39 year old men. A higher BMI was associated with persistent knee/hip pain in 40–59 year old women, and 50–59 year old men. Eight hundred sixty seven respondents completed the second questionnaire. Knee/hip injuries and surgeries were more common in individuals with prolonged persistent knee than hip pain.

**Conclusions:**

Knee/hip pain within the last month was frequent among individuals aged 29–59 years. Multiple known osteoarthritis risk factors were associated with presence of knee/hip pain. Joint injury and previous surgery were more common in individuals with knee than hip pain. The results support the notion that joint injury and overweight during early adulthood are signs of a trajectory towards symptomatic osteoarthritis later in life and may help earlier identification of groups at high risk of future symptomatic osteoarthritis.

**Trial registration:**

ClinicalTrials.gov (NCT02797392). Registered April 29,2016.

## Background

Knee and hip osteoarthritis (OA) affect more than 235 million people worldwide and is a major contributor to years lived with disability according to the WHO Global Burden of Disease Study [1]. Important risk factors for knee and hip OA include age, obesity, and female gender [2–4], but recent studies also suggest that physical inactivity may play a role [5]. In addition, biomechanical factors such as malalignment and occupational physical exposure are considered to modify the risk of OA development by altering joint loading [6–9].

Risk factors for OA development are usually studied in older high-risk populations, as the time to develop OA is usually long. Knowledge on the presence of OA risk factors, and in particular modifiable risk factors, in younger populations could help earlier identification of individuals at high risk of developing OA and may offer opportunity to prevent or delay the development of OA [10].

Few attempts have been made to investigate the presence of OA risk factors in early life. A recent narrative review reported only few studies assessing childhood or early adulthood risk factors for later knee OA development [11]. High BMI was found to be independently related to OA in later life, whereas limited evidence from mainly retrospective studies suggested that injury, childhood malalignment, socioeconomic status and physical abuse are associated with OA later in life [8, 11–14]. It is difficult to perform longitudinal studies addressing early adulthood risk factors for OA. Joint pain is a cardinal symptom of OA, which is often present before structural changes are visible on radiographs [15]. Given the strong link between persistent knee/hip pain and later development of OA, younger patients with knee and hip pain may serve a useful model to study the potential presence of OA risk factors earlier in life [11, 16].

The aims of this two stage cross-sectional study were: a) to investigate the prevalence of risk factors for knee and hip OA in a population of 29 to 59 year old individuals, b) to estimate the magnitude and direction of association between risk factors for knee and hip OA and presence of persistent knee and/or hip pain in a population of 29 to 59 year old individuals, and c) to investigate the prevalence of risk factors for knee and hip OA, including specific biomechanical risk factors, in a population of 29 to 59 year old individuals with prolonged persistent knee or hip pain, respectively.

## Methods

### Design

This was a two-stage cross-sectional survey. In the first stage we surveyed people participating in the “Early detection and prevention” (TOF) pilot study. In the second stage individuals who reported knee and/or hip problems at the first stage survey received a second survey with additional detailed questions. All data were self-reported via online surveys.

### Stage I: TOF pilot study

The TOF study is a population-based study on residents from the Region of Southern Denmark [17]. Almost all Danish residents are listed with a general practitioner (GP) [18]. During the course of one year about 85% of all residents are in contact with their GP [19]. The purpose of the TOF study is to early and systematically identify citizens with health risk behaviour and a high risk of lifestyle related diseases, and to offer targeted and coherent prevention in the primary care sector. In 2016 a pilot study (TOF pilot study) was performed to test the feasibility of the intervention.

All GPs (*n* = 68) in the municipalities of Haderslev and Varde (Denmark) were invited to participate in the TOF pilot study. Of these, 47 GPs agreed to participate. Two hundred citizens per GP were randomly selected among patients born between 1957 and 1986. Selection procedures allowed for simultaneous selection of citizens living together and resulted in a source population of 9400 citizens. Information on age and gender for all citizens was extracted from the personal identification numbers. All selected citizens received an invitation and were asked to sign an electronic consent form if they wished to participate in the study.

A survey including 15 items was mailed to all consenting participants. The survey included questions on health risk behaviour for lifestyle diseases, symptoms of Chronic Obstructive Lung Disease (COPD) and questions on possible risk factors for OA such as BMI, the presence of persistent knee and/or hip pain, previous knee/hip injury, previous knee/hip surgery, leisure time physical activity, and family history of OA.

#### Knee/hip pain vs. knee/hip problems

**Fig. 1:**
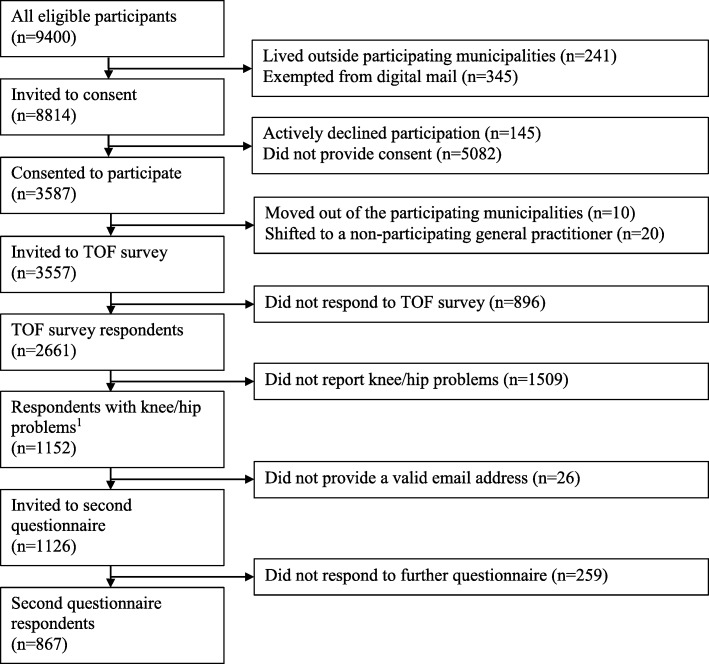
Flow diagram showing participants of the two-stage cross-sectional survey ^1^ Presence of knee and/or hip problems was defined as having knee and/or hip pain, a previous knee/hip injury or a knee/hip surgery reported in the TOF pilot study

The outcome of interest was persistent knee and/or hip pain. This was evaluated by the statement “I have had persistent pain or discomfort in my knees or hips during the last month”, response options: yes/no. A comparable question has previously been used although we adjusted the question to focus on persistent pain during the last month rather than 12 months [20]. Participants reported if they had had an injury to their knees or hips that caused them to visit a doctor, and if they had had previous surgery to their knee (s) and/or hip (s). The item regarding injury was adapted from The Knee Pain Screening Tool (KNEST) and has demonstrated good completion and test-retest reliability in 50+ year-olds on postal administration (> 90% agreement) [21]. The item regarding knee and/or hip surgery was adapted from the Osteoarthritis Initiative [22]. Presence of knee and/or hip *problems* was defined as having persistent knee and/or hip pain, a previous knee/hip injury and/or a previous knee/hip surgery. The assessment of the presence of knee and/or hip problems was only used to select TOF pilot study responders who should receive the second questionnaire on knee/hip-relevant risk factors (Fig. 1).

#### Leisure time physical activity

Leisure time physical activity was evaluated by the question “If you think of the past year, which of the following describes your recreational activities best?”, response options: “I read, watch TV or do other sedentary activities” (sedentary), “I walk, cycle, or perform other light physical activities at least 4 hours a week” (low intensity activities), “I do recreational sports or perform heavy gardening or alike at least 4 hours a week” (moderate intensity activities) and “I do intense exercise several times a week and participate in sport competitions regularly” (high intensity activities). This question has been used in several epidemiological studies [23–25].

#### Family history of OA

Family history of OA was evaluated by the question “Have any of your closest relatives – before the age of 70 years – had osteoarthritis? (closest relatives are grandparents, parents, siblings, children)”, with the following answer options: “no”, “yes, one person”, “yes, two or more” or “I don’t know”. Both “yes, one person” and “yes, two or more” were categorized as having a family history of OA. This question was modified to osteoarthritis from a question regarding family history of diabetes [24].

### Stage II: Second questionnaire on knee/hip-relevant risk factors

Participants reporting knee and/or hip problems in the TOF pilot study survey received a second questionnaire that included questions about the most symptomatic joint (i.e. left/right knee or hip), knee alignment, foot rotation, and physical occupational exposures. This second survey consisted of single item questions used in different questionnaires regarding the risk of osteoarthritis [8, 26] and was sent out in two mailings. The first mailing was distributed to the early responders to the first survey (*n* = 1030) 6 weeks after the first survey was sent out. The second mailing was distributed to the late responders (*n* = 96; participants who replied to the first survey after 6–14 weeks) 14 weeks after the first survey was distributed.

#### Knee alignment and foot rotation

Patients were asked to grade knee alignment and foot rotation by using a diagram that illustrated the direction and severity of each alignment grade. This was done separately for both knees and feet. For knee alignment, both answer options for varus alignment (“very bow legged” and “bow legged”) were combined, and the same was done for valgus alignment (knock-kneed). For foot rotation, answer options for feet rotated out (“very turned out feet” and “turned out feet”) and feet rotated in (“very turned in feet” and “turned in feet”) were also combined. These questions have demonstrated good reproducibility (κ = 0.73 and 0.87) and excellent validity [27], and have been used in epidemiological studies [8].

#### Occupational physical exposures

Occupational physical exposures were evaluated by the question “For the job or occupation you had for the longest time, did you do any of the following nearly every day?” with the following answer options: “bending for 2 hours or more”, “walking for 2 hours or more over level ground”, “kneeling for 30 minutes or more”, “squatting for 30 minutes or more”, “climbing a total of 5 or more flights of stairs”, “lifting or moving objects of 10 kg or heavier”, “driving a car for 4 hours or more”, “none of the above”. This question has previously been used in interviews and epidemiological studies [26, 28].

### Statistical analysis

Descriptive statistics are reported as means with standard deviation (SD) or numbers with proportions (%) as appropriate. Adjusted response rates were calculated corresponding to the American Association for Public Opinion Research’s (AAPOR) [29]. We stratified the analysis on prevalence of persistent knee and/or hip pain and OA risk factors for women and men in different age groups (29–39, 40–49 and 50–59 years old). Logistic regression using complete case analysis was used to estimate the magnitude and direction of the association between persistent knee and/or hip pain and OA risk factors. BMI was treated as a continuous variable, and individuals reporting high and moderate intensity activities were combined into one category in logistic regression analyses due to a low number of participants reporting high intensity activities. Risk ratios (RR) with 95% confidence intervals were estimated from logistic regressions using the method described by Norton et al. [30] and were expressed as crude and adjusted (i.e. including all variables in the model) RR for both women and men stratified by age groups. Model checking was performed with Hosmer-Lemeshow tests, linktests and visual inspection of residual plots. The presence of prolonged persistent knee and/or hip pain was defined as persistent knee and/or hip pain reported at both the first and second stage of this survey. We described the prevalence of OA risk factors, including specific biomechanical factors, in people with prolonged persistent knee pain and hip pain separately for both women and men. Data from the leg with the most symptomatic joint (index leg) was used for analyses for knee alignment and foot rotation. All analyses were performed using STATA version 15, with an alpha level of 0.05 or less considered as statistically significant.

## Results

### Cohort recruitment

#### Stage I: TOF pilot study

From the source population of 9400 citizens, 8814 citizens received an invitation to participate in the study (586 citizens lived outside the participating municipalities, or were exempted from digital mail) (Fig. 1 and Table 1). A total of 3587 citizens provided informed consent. Of consenting participants, 30 moved out of the participating municipalities or shifted to a non-participating GP before being invited to fill in the survey. Thus, 3557 citizens received the survey in September 2016, of whom 2661 (75%) answered the survey, giving an adjusted response rate of 30% (Table 1). Of the 2661 responders, 1152 (43%) reported that they had experienced knee and/or hip problems (i.e. reporting persistent knee and/or hip pain or discomfort in the last month, or having had a knee/hip injury or surgery). These individuals were eligible for the second questionnaire.

**Table 1:** Age, sex, persistent pain and risk factors at different study phases

	Source population	Invited to consent	Consented to participate in TOF pilot study	Invited to respond to TOF survey	TOF survey respondents	TOF survey respondents with knee/hip problems ^a^	Invited to respond to second questionnaire	Second questionnaire respondents
*N*	9400	8814	3587	3557	2661	1152	1126	867
Age (years)								
29–39	2633 (28)	2488 (28)	669 (19)	665 (19)	430 (16)	165 (14)	159 (14)	116 (13)
40–49	3179 (34)	2989 (34)	1147 (32)	1136 (32)	811 (30)	326 (28)	322 (29)	236 (27)
50–59	3588 (38)	3337 (38)	1771 (49)	1756 (49)	1420 (53)	661 (57)	645 (57)	515 (59)
Sex								
Women	4644 (49)	4379 (50)	1982 (55)	1964 (55)	1497 (56)	638 (55)	629 (56)	476 (55)
Men	4756 (51)	4435 (50)	1605 (45)	1593 (45)	1164 (44)	514 (45)	497 (44)	391 (45)
Persistent knee/hip pain					729 (27)	729 (63)	714 (63)	561 (65)
BMI^b^								
Normal/underweight					1102 (41)	420 (36)	409 (36)	310 (36)
Overweight					1001 (38)	444 (39)	433 (38)	335 (39)
Obesity					558 (21)	288 (25)	284 (25)	222 (26)
Leisure time physical activity								
High intensity activities					66 (2)	26 (2)	23 (2)	14 (2)
Moderate intensity activities					763 (29)	332 (29)	324 (29)	253 (29)
Low intensity activities					1448 (54)	626 (54)	615 (55)	484 (56)
Sedentary					384 (14)	168 (15)	164 (15)	116 (13)
Previous knee/hip injury					737 (28)	737 (64)	718 (64)	559 (64)
Previous knee/hip surgery					395 (15)	395 (34)	388 (34)	305 (35)
Family history of osteoarthritis								
No					999 (38)	304 (26)	296 (26)	222 (26)
I don’t know					494 (19)	217 (19)	215 (19)	168 (19)
Yes					1168 (44)	631 (55)	615 (55)	477 (55)

Figures represent numbers and percentages unless otherwise stated

*BMI* body mass index

^a^Knee/hip problems = defined as the participants who reported to have had persistent knee and/or hip pain or discomfort during the last month, or that they had had a knee/hip injury or surgery

^b^Normal/underweight (BMI ≤ 25), overweight (25 > BMI ≤ 30), obesity (BMI > 30)

More women and older citizens agreed to participate (Table 1). Responders with knee and/or hip problems more often reported a family history of OA, compared to all responders of the first questionnaire.

#### Stage II: Second questionnaire on hip/knee-relevant risk factors

During the two mailings (October and December 2016) of the second questionnaire, 26 citizens were excluded, as they had not provided a valid email address. Of the eligible study population of 1126 citizens, 867 (77%) responded to the second questionnaire. Fourteen of the responders did not complete the entire questionnaire.

### Knee pain

Of the 2661 responders to the first questionnaire, 729 (27%) reported to have persistent knee and/or hip pain (Table 1). The prevalence of persistent knee and/or hip pain was slightly higher among women (29%) than men (26%) (Table 2). The one-month prevalence increased with age in women (29–39 years: 22%, 40–49 years: 28%, 50–59 years: 31%), while in men persistent knee and/or hip pain seemed to be similar for the first two age groups (29–39 years: 23%, 40–49 years: 20%) and be higher in the oldest group (50–59 years: 30%).

**Table 2:** Prevalence of persistent knee/hip pain and risk factors for knee/hip osteoarthritis (*n* = 2661)

	Women		Men	
	Knee/hip pain	No knee/hip pain	Knee/hip pain	No knee/hip pain
29–39 years	54 (22)	196 (78)	41 (23)	139 (77)
BMI (kg/m^2^), mean (SD)	27.4 (6.3)	26.4 (6.3)	26.4 (4.8)	26.2 (3.9)
Leisure time physical activity				
High intensity activities	1 (2)	6 (3)	2 (5)	12 (9)
Moderate intensity activities	10 (19)	43 (22)	14 (34)	43 (31)
Low intensity activities	29 (54)	114 (58)	17 (41)	57 (41)
Sedentary	14 (26)	33 (17)	8 (20)	27 (19)
Previous knee/hip injury	27 (50)	32 (16)	21 (51)	34 (24)
Previous knee/hip surgery	11 (20)	17 (9)	10 (24)	14 (10)
Family history of osteoarthritis				
No	6 (11)	72 (37)	13 (32)	58 (42)
I don’t know	12 (22)	38 (19)	9 (22)	30 (22)
Yes	36 (67)	86 (44)	19 (46)	51 (37)
40–49 years	130 (28)	331 (72)	69 (20)	281 (80)
BMI (kg/m^2^), mean (SD)	29.1 (7.6)	26.1 (5.4)	28.5 (4.8)	27.0 (4.7)
Leisure time physical activity				
High intensity activities	2 (2)	6 (2)	1 (1)	9 (3)
Moderate intensity activities	34 (26)	94 (28)	24 (35)	123 (44)
Low intensity activities	67 (52)	184 (56)	30 (43)	111 (40)
Sedentary	27 (21)	47 (14)	14 (20)	38 (14)
Previous knee/hip injury	61 (47)	49 (15)	35 (51)	65 (23)
Previous knee/hip surgery	24 (18)	29 (9)	19 (28)	45 (16)
Family history of osteoarthritis				
No	29 (22)	128 (39)	18 (26)	138 (49)
I don’t know	22 (17)	53 (16)	15 (22)	42 (15)
Yes	79 (61)	150 (45)	36 (52)	101 (36)
50–59 years	245 (31)	541 (69)	190 (30)	444 (70)
BMI (kg/m^2^), mean (SD)	27.6 (5.1)	26.0 (5.1)	28.2 (4.7)	26.9 (4.0)
Leisure time physical activity				
High intensity activities	2 (1)	9 (2)	2 (1)	14 (3)
Moderate intensity activities	47 (19)	121 (22)	57 (30)	153 (34)
Low intensity activities	161 (66)	351 (65)	105 (55)	222 (50)
Sedentary	35 (14)	60 (11)	26 (14)	55 (12)
Previous knee/hip injury	101 (41)	105 (19)	111 (58)	96 (22)
Previous knee/hip surgery	52 (21)	51 (9)	60 (32)	63 (14)
Family history of osteoarthritis				
No	50 (20)	207 (38)	53 (28)	227 (51)
I don’t know	40 (16)	101 (19)	48 (25)	84 (19)
Yes	155 (63)	233 (43)	89 (47)	133 (30)

Figures represent numbers and percentages unless otherwise stated

*BMI* body mass index

In total, 416 participants reported to have prolonged persistent knee and/or hip pain (i.e. reporting knee and/or hip pain both at the first and second stage of this survey) (Table 4). Of this group with prolonged persistent pain, 142 women reported knee pain and 102 reported hip pain while 123 men reported knee pain and 49 reported hip pain.

### Association between OA risk factors and knee/hip pain

Table 2 describes the prevalence of knee and/or hip pain and OA risk factors stratified by age and sex, and shows that women more often reported a family history of OA than men (49% vs. 37%). Only 2% of participants reported “high intensity” physical activity level.

In multivariable analyses, previous knee and/or hip injury was associated with persistent knee and/or hip pain for both genders and in all age groups (Table 3). In the younger age group (29–39 years), the risk ratio (95% CI) for previous knee and/or hip injury of having persistent knee and/or hip pain was higher for women than men (3.05 (1.84–5.05) vs. 2.34 (1.23–4.47)), whereas it was the opposite in the older age group (50–59 years old; 1.64 (1.31–2.06) vs. 2.61 (1.97–3.45)). Having a family history of OA was also associated with persistent knee and/or hip pain in all analyses, except for younger men (1.31 (0.71–2.44)). Higher BMI was associated with persistent knee and/or hip pain in 40–49 and 50–59 year old women (1.06 (1.02–1.10) and 1.04 (1.01–1.08)), and in 50–59 year old men (1.04 (1.00–1.09). The observed effect of BMI on risk of knee and/or hip pain was a 4 and 6% increase in risk per point increase in BMI, respectively. Having had previous knee and/or hip surgery and the level of leisure time physical activity were factors not associated with persistent knee and/or pain in any of the adjusted analyses.

**Table 3:** Association of risk factors for hip/knee osteoarthritis with persistent knee/hip pain (*n* = 2661)

	Women		Men	
	Univariate RR (95% CI)	Adjusted^a^ RR (95% CI)	Univariate RR (95% CI)	Adjusted^a^ RR (95% CI)
29–39 years	*n* = 250		*n* = 180	
BMI (kg/m^2^)	1.02 (0.98–1.07)	1.00 (0.96–1.03)	1.01 (0.94–1.08)	1.02 (0.94–1.10)
Leisure time physical activity				
High and moderate intensity activities	Reference	Reference	Reference	Reference
Low intensity activities	1.11 (0.59–2.07)	1.22 (0.68–2.20)	1.02 (0.56–1.86)	1.21 (0.67–2.19)
Sedentary	1.62 (0.81–3.24)	1.48 (0.75–2.93)	1.01 (0.48–2.14)	1.28 (0.61–2.68)
Previous knee/hip injury	**3.24 (2.07–5.06)**	**3.05 (1.84–5.05)**	**2.39 (1.41–4.03)**	**2.34 (1.23–4.47)**
Previous knee/hip surgery	**2.03 (1.19–3.46)**	0.85 (0.43–1.71)	**2.10 (1.19–3.70)**	1.22 (0.57–2.62)
Family history of osteoarthritis				
No	Reference	Reference	Reference	Reference
I don’t know	**3.12 (1.25–7.78)**	2.41 (1.00–5.84)	1.26 (0.59–2.68)	1.39 (0.68–2.84)
Yes	**3.84 (1.70–8.68)**	**2.92 (1.32–6.45)**	1.48 (0.79–2.77)	1.31 (0.71–2.44)
40–49 years	*n* = 461		*n* = 350	
BMI (kg/m^2^)	**1.07 (1.04–1.11)**	**1.06 (1.02–1.10)**	**1.06 (1.00–1.11)**	1.04 (0.99–1.10)
Leisure time physical activity				
High and moderate intensity activities	Reference	Reference	Reference	Reference
Low intensity activities	1.01 (0.71–1.43)	0.85 (0.62–1.15)	1.34 (0.83–2.16)	1.32 (0.83–2.09)
Sedentary	1.38 (0.91–2.08)	0.99 (0.66–1.48)	1.69 (0.95–3.00)	1.45 (0.80–2.63)
Previous knee/hip injury	**2.82 (2.15–3.69)**	**2.73 (2.01–3.70)**	**2.57 (1.70–3.88)**	**2.55 (1.57–4.14)**
Previous knee/hip surgery	**1.74 (1.24–2.44)**	0.87 (0.56–1.37)	**1.70 (1.08–2.67)**	0.87 (0.49–1.55)
Family history of osteoarthritis				
No	Reference	Reference	Reference	Reference
I don’t know	1.59 (0.98–2.57)	1.44 (0.93–2.23)	**2.28 (1.23–4.22)**	**2.01 (1.10–3.68)**
Yes	**1.87 (1.28–2.71)**	**1.53 (1.09–2.15)**	**2.28 (1.36–3.82)**	**2.00 (1.20–3.29)**
50–59 years	*n* = 786		*n* = 634	
BMI (kg/m^2^)	**1.05 (1.02–1.09)**	**1.04 (1.01–1.08)**	**1.07 (1.03–1.12)**	**1.04 (1.00–1.09)**
Leisure time physical activity				
High and moderate intensity activities	Reference	Reference	Reference	Reference
Low intensity activities	1.15 (0.88–1.51)	1.08 (0.83–1.39)	1.23 (0.94–1.61)	1.19 (0.93–1.53)
Sedentary	1.35 (0.94–1.92)	1.11 (0.77–1.59)	1.23 (0.84–1.81)	1.18 (0.82–1.69)
Previous knee/hip injury	**1.97 (1.62–2.41)**	**1.64 (1.31–2.06)**	**2.90 (2.29–3.67)**	**2.61 (1.97–3.45)**
Previous knee/hip surgery	**1.79 (1.43–2.24)**	1.20 (0.90–1.60)	**1.92 (1.52–2.42)**	0.97 (0.72–1.31)
Family history of osteoarthritis				
No	Reference	Reference	Reference	Reference
I don’t know	**1.46 (1.02–2.09)**	1.33 (0.94–1.88)	**1.92 (1.38–2.68)**	**1.57 (1.15–2.14)**
Yes	**2.05 (1.56–2.71)**	**1.76 (1.34–2.31)**	**2.12 (1.58–2.83)**	**1.63 (1.23–2.15)**

^a^Adjusted for BMI, leisure time physical activity, previous knee/hip injury, previous knee/hip surgery and family history of osteoarthritis

*RR* Risk Ratio, *BMI* body mass index

Figures in bold represent statistical significant effects (*p* < 0.05)

### OA risk factors in people with prolonged persistent knee or hip pain, respectively

Both women and men with prolonged persistent knee pain more often reported joint injury (women 60%, men 64%) and joint surgery (women 36%, men 37%) than individuals with prolonged persistent hip pain (injury: women 37%, men 51%; surgery: women 12%, men 18%) (Table 4). Men with prolonged persistent knee and hip pain more often reported to be occupationally physically exposed than women. The prevalence of the other risk factors was similar among men and women with knee and hip pain.

**Table 4:** Prevalence of risk factors for knee/hip osteoarthritis in people with prolonged persistent knee and hip pain

	Women		Men	
	Knee pain	Hip pain	Knee pain	Hip
	*n* = 142^a^	*n* = 102^a^	*n* = 123	*n* = 49
Age (years), mean (SD)	50.6 (6.6)	49.2 (7.7)	49.4 (7.7)	52.6 (6.7)
BMI (kg/m^2^), mean (SD)	28.4 (6.0)	27.3 (5.7)	28.1 (5.0)	28.5 (4.8)
Leisure time physical activity				
High intensity activities	2 (1)	0 (0)	3 (2)	0 (0)
Moderate intensity activities	33 (23)	22 (22)	34 (28)	13 (27)
Low intensity activities	85 (60)	67 (66)	65 (53)	26 (53)
Sedentary	22 (15)	13 (13)	21 (17)	10 (20)
Previous knee/hip injury	85 (60)	38 (37)	79 (64)	25 (51)
Previous knee/hip surgery	51 (36)	12 (12)	46 (37)	9 (18)
Family history of osteoarthritis				
No	29 (20)	18 (18)	30 (24)	12 (24)
I don’t know	23 (16)	20 (20)	31 (25)	11 (22)
Yes	90 (63)	64 (63)	62 (50)	26 (53)
Knee alignment				
Varus	9 (6)	4 (4)	14 (11)	5 (10)
Neutral	110 (79)	84 (83)	102 (83)	43 (88)
Valgus	21 (15)	13 (13)	7 (6)	1 (2)
Foot rotation				
Feet rotated out	44 (31)	30 (30)	59 (48)	20 (41)
Straight	80 (57)	62 (61)	62 (50)	27 (55)
Feet rotated in	16 (11)	9 (9)	2 (2)	2 (4)
Occupational physical exposures				
Bending for 2 h or more	33 (24)	29 (29)	28 (23)	14 (29)
Walking for 2 h or more over level ground	67 (48)	46 (46)	71 (58)	29 (59)
Kneeling for 30 min or more	21 (15)	10 (10)	41 (33)	16 (33)
Squatting for 30 min or more	18 (13)	6 (6)	21 (17)	9 (18)
Climbing a total of 5 or more flights of stairs	14 (10)	4 (4)	20 (16)	9 (18)
Lifting or moving objects of 10 kg or heavier	36 (26)	21 (21)	57 (46)	32 (65)
Driving a car for 4 h or more	7 (5)	5 (5)	21 (17)	9 (18)
None of the above	51 (36)	41 (41)	25 (20)	8 (16)

Reported for the populations with prolonged persistent knee and hip pain (*n* = 416)

Figures represent numbers and percentages unless otherwise stated

^a^Some data is missing for the risk factors knee alignment, foot rotation and occupational physical exposures (women with knee pain: *n* = 140; women with hip pain: *n* = 101)

## Discussion

In this population-based sample of individuals aged 29 to 59 years, persistent knee and/or hip pain within the last month was reported by more than 20% of participants, including those in the younger age groups. We found that typical OA risk factors such as previous knee/hip injury, a family history of OA and higher BMI were associated with persistent knee and/or hip pain in 50–59 year old men and women. Furthermore, in 40–49 year old men and women, previous knee/hip injury and a family history of OA were associated with pain, while higher BMI was associated with pain only in women of this age. In addition, in younger men and women aged 29–39 years, previous knee/hip injury was associated with pain, while a family history of OA was associated with pain in younger women only. Finally, we found that injuries and surgeries were more common in people with prolonged persistent knee than hip pain, while men with prolonged persistent pain were more often physically exposed in their occupation than women.

In this study, we investigated the prevalence of knee and/or hip pain and commonly known OA risk factors in a population aged 29–59 years (mean age 48.8 (SD 7.8)). Most previous studies have focused on OA risk factors in older populations [15, 31]. Despite pain usually being more common with increasing age, we found persistent knee and/or hip pain within the previous month to be prevalent in a substantial part of younger adults (i.e. 22% of 29–39 year olds and in 25% of 40–49 year olds), which is consistent with other studies [32]. Given the link between persistent knee/hip pain and later development of OA, we considered it a useful way to investigate if typical risk factors of OA were also prevalent at younger ages and to determine their association with persistent pain [11, 12].

The observed presence of modifiable OA risk factors (e.g. obesity and joint injury) in the younger age groups is an important finding, as it highlights factors that can potentially be targeted with weight management and injury prevention programs [10, 11]. Further, primary health care professionals should also consider these factors when younger patients present in the clinic with joint pain, as it may help motivating patients to engage in secondary prevention interventions such as exercise therapy and/or weight management to prevent or postpone OA development [10].

Joint injury was found to be associated with knee and/or hip pain across all age groups. Typically, an injury results in pain and functional loss, and will in some cases be treated with surgery. Thus, the group of participants that reported an injury was nearly identical to the group that reported surgery. This likely explains the lack of association between pain and surgery in the multivariable models, while significant associations for both injury and surgery were observed in univariate analyses. Higher prevalence’s of injury and surgery were found in people with prolonged persistent knee pain than in those with hip pain. This may reflect that knee injury is more common in sports than hip injury [14]. In addition, an earlier study showed that a knee joint injury at younger age was associated with knee OA development, although none of the individuals with a hip injury (*n* = 13) developed hip OA [14].

The involvement of either the knee or hip joint could possibly explain more of our findings, such as the absence of an association between BMI and knee and/or hip pain in the youngest age groups. Several studies into OA risk factors have reported differences between risk factors for knee OA, and for hip OA [6, 33, 34]. In addition, studies have shown higher BMI at younger age to be associated with later knee OA development, whereas there seems to be no association with hip OA development [12, 13]. Thus, separating data for the knee and the hip joint could potentially be important.

Similarly, self-reported presence of varus knee, valgus knee, and toe-in/out angles during early adult life has been reported to be associated with increased risk of knee but not hip OA [8]. We reported the prevalence of malalignment separately for the population with prolonged knee pain and the population with prolonged hip pain. However, we could not assess the association of malalignment with prolonged knee and hip pain as malalignment was not assessed in individuals without knee/hip problems.

In our study, we did not find any association between leisure time physical activity level and knee/hip pain. Similar findings were reported in a study on adults aged 50 years or older (*n* = 1276) where the same percentage of individuals with and without knee pain met physical activity recommendations [35]. It should be noted, that only few people in all ages groups were involved in high-level physical activities in the present study.

Some limitations apply to this study. We had no information on the OA status of respondents. Thus, it is likely that some patients, particularly in the older age group, may have had structural and clinical symptoms consistent with OA. The time of onset of osteoarthritis is not a clear, unambiguous event and therefore the extent to which intervention targeted at modifiable risk factors present in this symptomatic population aged 29–59 years would represent primary or secondary prevention of osteoarthritis is necessary blurred. Given the known strong age-related increase in prevalence of osteoarthritis across this age range it is likely that as one moves towards the upper end of this age range interventions are increasingly likely to be on cases of early or established osteoarthritis. We did not distinguish between pain originating from the hip or the knee joint in the first stage of the survey. This was only specified in the second stage of the survey including participants reporting knee/hip problems in the first survey. Similarly, prolonged persistent pain was also first recorded in the second stage of the survey, which is the reason for only reporting the prevalence of OA risk factors in this subgroup. All data in the present study were self-reported with inherent risk of recall bias, and possibly over- or underreporting of pain and OA risk factors. Recall error might also explain why the prevalence of injury did not increase by age as expected. Family information bias (i.e. better recall of disease presence in family members when this person suffers from the disease) or women more often being involved in social relations might explain the higher prevalence of family history of OA in women versus men. In addition, selection bias could also be present because not all invited citizens responded to the survey, and individuals who did respond were more often older and women. Lastly, the psychometric properties have not been evaluated for all the questions used.

## Conclusions

In this population-based study, knee and hip pain were found in more than 20% of participants, even in the youngest age group of 29–39 year olds where the prevalence of established radiographic OA is known to be low. In addition, multiple known risk factors for knee and hip OA, such as higher BMI, were found to be associated with the presence of knee and/or hip pain. Joint injuries and surgeries were more prevalent in individuals with knee than hip pain. This information supports the notion that joint injury and overweight during early adulthood are signs of a trajectory towards symptomatic osteoarthritis later in life and may help earlier identification of groups at high risk of future symptomatic osteoarthritis.

## Abbreviations

AAPOR: American Association for Public Opinion Research
BMI: body mass index
COPD: Chronic Obstructive Lung Disease
GP: general practitioner
KNEST: The Knee Pain Screening Tool
OA: osteoarthritis
RR: risk ratio
TOF pilot study: “Early detection and prevention” pilot study
